# Measuring ventilation and modelling M. tuberculosis transmission in indoor congregate settings, rural KwaZulu-Natal

**DOI:** 10.5588/ijtld.16.0085

**Published:** 2016-07-08

**Authors:** J. G. Taylor, T. A. Yates, M. Mthethwa, F. Tanser, I. Abubakar, H. Altamirano

**Affiliations:** * University College London (UCL) Institute for Environmental Design and Engineering, Bartlett School of Environment, Energy and Resources, UCL, London, UK; † Wellcome Trust Africa Centre for Population Health, Mtubatuba, South Africa; ‡ Centre for Infectious Disease Epidemiology, Research Department of Infection and Population Health, UCL, London, UK; § School of Nursing and Public Health, University of KwaZulu-Natal, Durban, South Africa; ¶ Centre for the AIDS Programme of Research in South Africa (CAPRISA), University of KwaZulu Natal, Congella, South Africa; # Institute for Global Health, UCL, London, UK

**Keywords:** Wells-Riley equation, infection control, South Africa

## Abstract

SETTING: Molecular epidemiology suggests that most Mycobacterium tuberculosis transmission in high-burden settings occurs outside the home.

OBJECTIVE: To estimate the risk of M. tuberculosis transmission inside public buildings in a high TB burden community in KwaZulu-Natal, South Africa.

DESIGN: Carbon dioxide (CO_2_) sensors were placed inside eight public buildings. Measurements were used with observations of occupancy to estimate infection risk using an adaptation of the Wells-Riley equation. Ventilation modelling using CONTAM was used to examine the impact of low-cost retrofits on transmission in a health clinic.

RESULTS: Measurements indicate that infection risk in the church, classroom and clinic waiting room would be high with typical ventilation, occupancy levels and visit durations. For example, we estimated that health care workers in a clinic waiting room had a 16.9–24.5% annual risk of M. tuberculosis infection. Modelling results indicate that the simple addition of two new windows allowing for cross-ventilation, at a cost of US$330, would reduce the annual risk to health care workers by 57%.

CONCLUSIONS: Results indicate that public buildings in this community have a range of ventilation and occupancy characteristics that may influence transmission risks. Simple retrofits may result in dramatic reductions in M. tuberculosis transmission, and intervention studies should therefore be considered.

## RESUME

CONTEXTE : L'épidémiologie moléculaire suggère que la majorité de la transmission de Mycobacterium tuberculosis dans les zones durement touchées survient hors du domicile.

OBJECTIF : Estimer le risque de transmission de M. tuberculosis à l'intérieur de bâtiments publics dans une communauté lourdement frappée par la TB, KwaZulu-Natal, Afrique du Sud.

SCHEMA : Des capteurs de dioxyde de carbone (CO_2_) ont été placés dans huit bâtiments publics. Les mesures réalisées ont permis grace à l'observation de l'occupation des lieux d'estimer le risque d'infection avec une adaptation de l'équation de Wells-Riley. Une modélisation de la ventilation utilisant CONTAM a permis d'examiner l'impact de renovations à faible coût sur la transmission dans un centre de santé.

RESULTATS : Les mesures ont montré que le risque d'infection dans l'église, la salle de classe et la salle d'attente du centre de santé seraient élevées avec une ventilation, un niveau d'occupation et une durée de présence typiques. Nous avons par exemple estimé que le personnel de santé dans une salle d'attente avait un risque annuel de 16,9% à 24,5% d'infectionà M. tuberculosis. Les résultats de la modélisation ont indiqué que la simple addition de deux nouvelles fenêtres permettant une ventilation croisée, pour un coût de US$330, réduirait le risque annuel pour le personnel de santé de 57%.

CONCLUSIONS : Les résultats montrent que les bâtiments publics dans cette communauté ont un ensemble de caractéristiques de ventilation et d'occupation qui pourraient influencer les risques de transmission. Des rénovations simples pourraient aboutir à une réduction importante de la transmission de M. tuberculosis et il faudrait envisager des études d'intervention.

## RESUMEN

MARCO DE REFERENCIA: Los datos de epidemiología molecular indican que la mayor parte de la transmisión de Mycobacterium tuberculosis en los entornos con alta carga de morbilidad por tuberculosis ocurre por fuera del hogar.

OBJETIVO: Estimar el riesgo de transmisión de M. tuberculosis dentro de los edificios públicos en una comunidad con alta carga de morbilidad, en KwaZulu-Natal, Africa del Sur.

MÉTODO: Se dispusieron sensores de dióxido de carbono al interior de ocho edificios públicos. Las mediciones permitieron evaluar el grado de ocupación de los recintos, con el objeto de estimar el riesgo de infección mediante una adaptación de la ecuación de Wells-Riley. Se creó una modelización del sistema de ventilación con la herramienta CONTAM con el propósito de evaluar la repercusión de la modernización a bajo costo en la transmisión en un consultorio.

RESULTADOS: Las mediciones revelaron que el riesgo de infección en una iglesia, un salón de clase y la sala de espera se un consultorio sería alto considerando la ventilación, la tasa de ocupación y la duración de la consulta habituales. Por ejemplo, se calculó que los profesionales de salud en la sala de espera de un consultorio tenían un riesgo anual de infección por M. tuberculosis de 16,9% a 24,5%. Los resultados de la modelización de los sistemas indicaron que la simple adición de dos nuevas ventanas que aporten ventilación cruzada, con un costo de 330 dólares, disminuiría un 57% el riesgo anual de los profesionales de la salud.

CONCLUSIÓN: Los resultados del presente estudio indican que los edificios públicos en esta comunidad presentan una serie de características de ventilación y tasas de ocupación que pueden incidir en los riesgos de transmisión. Ejecutar modernizaciones sencillas puede procurar disminuciones drásticas de la transmisión de M. tuberculosis y por lo tanto es preciso considerar la realización de estudios de intervención que evalúen su impacto.

TUBERCULOSIS (TB) is a leading cause of death globally, with Southern Africa disproportionately affected by the disease. To achieve TB control, countries in Southern Africa must address the high force of infection.^1^,^2^ Molecular epidemiology from high-burden settings,^3^,^4^ including South Africa,^5^,^6^ suggests that only a small proportion of TB is due to Mycobacterium tuberculosis transmission between members of the same household. This likely reflects a high risk of transmission outside the home rather than any attenuation in the risk of household transmission. These empirical findings are supported by models using social contact pattern data and Wells-Riley calculations. In a Cape Town township, South Africa, such models suggest 84% of M. tuberculosis transmission occurs outside the home,^7^ with most transmission predicted to occur in schools, workplaces and on public transport.

Other public spaces might also be important sites of transmission. Health care facilities in South Africa bring together infectious TB cases and individuals with human immunodeficiency virus (HIV) related immunosuppression, generating a high risk of transmission with progression to disease. High rates of undiagnosed TB have been observed in hospital in-patients in KwaZulu-Natal^8^ and in clinic attendees elsewhere in South Africa.^9^,^10^ Extensive transmission of drug-resistant M. tuberculosis has been described in KwaZulu-Natal health care facilities.^11^ Using qualitative observations, researchers in another high-burden South African community have speculated that drinking establishments and churches may be M. tuberculosis transmission ‘hot spots’.^12^

A recent systematic review found strong evidence of an association between building ventilation and transmission of airborne pathogens such as M. tuberculosis.^13^ Understanding ventilation in putative sites of transmission is important, both to estimate transmission risk and to evaluate the impact and feasibility of environmental modifications to limit M. tuberculosis transmission. One way in which transmission could be reduced is through a renewed focus on ventilation and on reducing the levels of ‘shared air’ to which individuals are exposed.^2^,^14^ Such environmental controls are advocated in TB infection control guidelines,^15^ but are often not implemented.^16^

Previous studies have estimated indoor M. tuberculosis transmission risk using the measured rate of decay of carbon dioxide (CO_2_) released inside buildings to estimate a ventilation rate, then incorporating this into the Wells-Riley equation.^17^,^18^ An alternative approach, proposed by Rudnick & Milton,^19^ uses the concept of ‘rebreathed air’. This method has been used to estimate rates of M. tuberculosis transmission in public transport in South Africa^20^ and in multiple buildings in a Cape Town township.^7^

The objective of the present paper is to estimate transmission risks in indoor congregate settings in KwaZulu-Natal, South Africa, and explore how they may be mitigated. We use Rudnick & Milton's approach to estimate M. tuberculosis transmission risk inside public buildings, both during a single visit and over a year. We then use the airflow analysis tool CONTAM (National Institute of Standards and Technology, Gaithersburg, MD, USA) to model ventilation and transmission risk in one such setting both before and after a number of improvements in ventilation.^21^

## METHODS

### Setting and building selection

**Figure 1:**
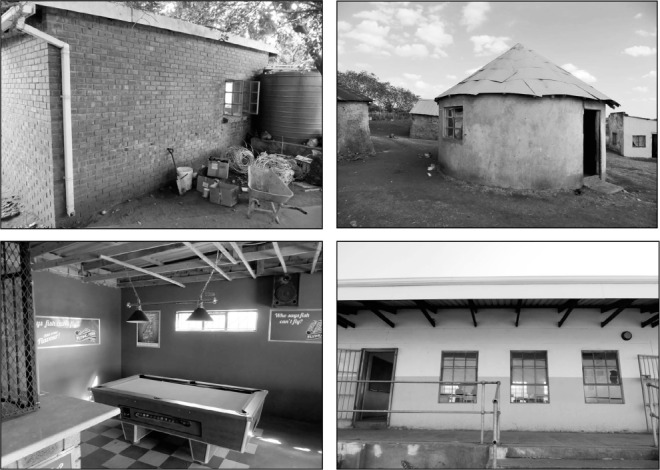
Photographs, clockwise from top left, of the clinic waiting room, the church, the high school classroom and the tavern.

This project was undertaken in Umkhanyakude District in KwaZulu-Natal. The district's TB notification rate was 878 per 100 000 population in 2013,^22^ and in 2011 29% of adults were HIV-positive.^23^ The study site comprised rural communities, a peri-urban township and a small market town. The buildings selected for study were a convenience sample of public spaces (Figure 1): a tavern, a bank, the waiting room in a social security office, a clinic waiting room, a large shop in the town, a small rural shop, a small rural church, a high school classroom, a post office and a fast-food restaurant. We were able to obtain the requisite permission for all public spaces, with the exception of the post office and the fast-food restaurant. Apart from the bank, which was mechanically ventilated, all of the buildings were naturally ventilated.

### Ethics

Approval was obtained from the University of KwaZulu-Natal Biomedical Research Ethics Committee (Durban, South Africa) before conducting this study (BE058/14). Written consent to take measurements and photographs was obtained from the custodian of each building.

### Sensor development and calibration

Low-cost battery-operated CO_2_ sensors were specially built for this project using a low-power non-dispersive infrared CO_2_ gas sensor (NDIR CO_2_ COZIR 0–5000 ppm sensor; accuracy: ±50 ppm ±3%).^24^ This was connected to an external HOBO U12 data logger (ONSET, Bourne, MA, USA), which also monitored temperature. The sensors were calibrated using ELTEK CO_2_ sensors (Haslingfield, UK) (0–5000 ppm, accuracy ±50 ppm).

### Physical measurements

**Figure 2:**
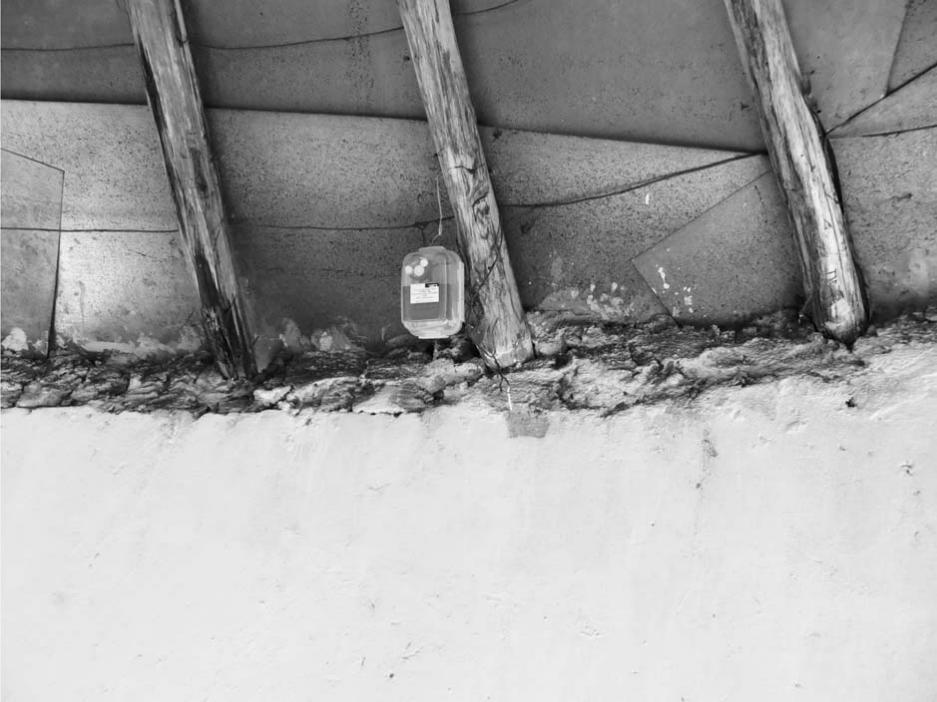
Carbon dioxide sensor mounted in the rafters of the church at approximately 2 m height.

A minimum of three sensors were located within each building. An additional sensor was placed immediately outside the building to record background concentrations of CO_2_. Sensors recorded concentrations of CO_2_ and temperature every minute for 10 days. In all indoor spaces, the only source of CO_2_ was occupant respiration; outdoor sensors were located as far from sources of CO_2_ as possible. An example of sensor placement can be seen in Figure 2.

### Measuring occupancy

We estimated transmission risk when buildings were in use. The number of occupants within the buildings was determined from discussions with building custodians (church, tavern), from building records (social security office, classroom, clinic waiting room) or by direct observation (large and small store, bank). The average length of stay was estimated following discussions with custodians and by direct observation (Appendix).^*^

### Data compilation and risk calculation

Data from the indoor sensors were averaged for each building to obtain the mean indoor concentrations of CO_2_ every minute. The equations proposed by Rudnick & Milton were used to calculate the M. tuberculosis transmission risk indoors (Appendix).^19^ We produced estimates assuming that one infectious individual was present, then with the number of infectors present estimated as a proportion of occupants. We assumed a prevalence of untreated active TB of 2% for the general population^25^ and 3.2% among those visiting the clinic.^9^ We then calculated risk per hour, and, using assumptions about visit duration and frequency, the risk per visit and over the course of a year. We also examined sensitivity of estimates to assumptions regarding infectiousness. Model parameters are presented in the Appendix.

### Modelling ventilation

To illustrate the potential reduction in M. tuberculosis transmission risk possible through building adaptations, we modelled ventilation in and feasible retrofits to the clinic waiting room using CONTAM 3.1.^21^ Estimated costs for the modelled retrofits were obtained from a local surveyor. Building measurements, observations of building operation and CO_2_ measurements informed model development. The model used a weather file representing average conditions for Durban, South Africa, with indoor temperature modelled as the mean monitored temperature (21.2°C). Wind exposures were modified for the local terrain. Simulations were run for a calendar year. The changes in ventilation rate and the consequent risk of M. tuberculosis transmission were then estimated for each building variant using the Wells-Riley equation.^26^

## RESULTS

**Figure 3:**
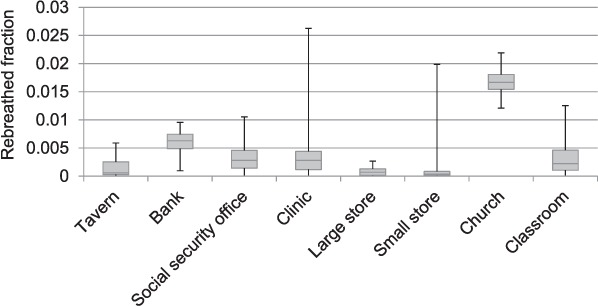
Ranges and interquartile ranges of the fraction of indoor air that was rebreathed air during the hours that the buildings were open to the public. These estimates were based on paired internal and external carbon dioxide measurements.

Data were collected between June and August 2014. The fraction of indoor air in each space that was exhaled breath during the hours the buildings were open to the public is presented in Figure 3. The results show that, during services, the church had the highest average fraction of rebreathed air (range 0.012–0.022), with the clinic waiting room briefly reaching the greatest fraction (0.026) for a 10-min period.

The estimated probabilities of an individual being infected with M. tuberculosis inside each building, both per hour of visit and during a visit of typical duration, are shown in Table 1. The probability of transmission was higher in the church and clinic than in other buildings, driven by both higher monitored CO_2_ levels and longer visit durations.

**Table 1:**
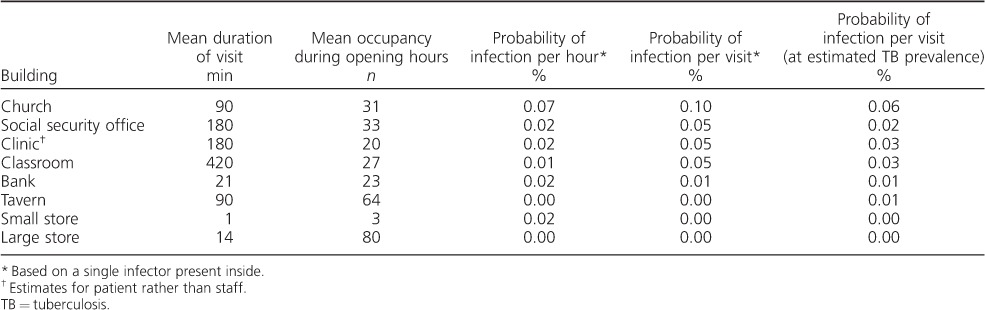
Estimated probabilities of an individual becoming infected with M. tuberculosis in the different monitored buildings

Estimates regarding the number of visits made to each location over the course of a year, and the corresponding annual risks, assuming a single infectious occupant and scaled for the estimated background TB prevalence, are given in Table 2. The classroom and church were estimated to have the highest risk, largely due to the frequency of visits, the length of time spent indoors and the number of occupants. The risk of transmission to health care workers was also predicted to be high.

**Table 2:**
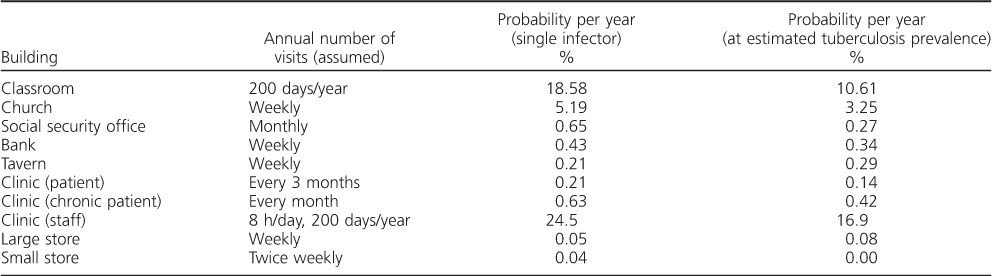
Estimated annual probabilities of acquiring M. tuberculosis infection in each public space

As might be expected, predicted transmission risks were highly dependent on assumptions regarding the rate at which persons with TB produced infectious ‘quanta’, particularly when extrapolated to estimate annual risk (Appendix).

### Ventilation modeling

Several low-cost potential building adaptations were modelled, including enlarging the area of the existing windows by 25%, the creation of two additional windows of the same size as the existing windows in locations that promote cross-ventilation and the installation of a mechanical extractor fan with a constant flow of 200 m^3^/h on the roof. We also modelled a more advanced adaptation involving the reconstruction of each wall with lattice brickwork from 1 m to 2 m in height, and open space from 2 m to 2.5 m, based on the design of the respiratory waiting room at the Hospital Nacional Sergio Bernales, Lima, Peru.^27^

**Figure 4:**
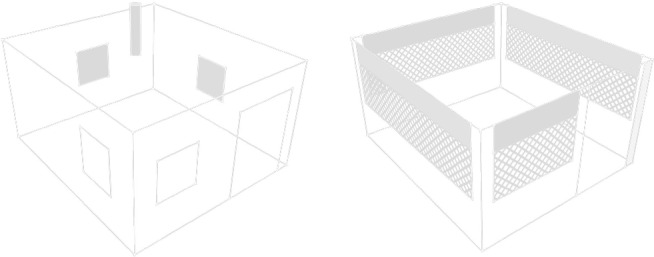
Clinic waiting room as modelled in CONTAM, with proposed retrofits. Low-cost retrofits (two additional windows or a chimney) can be seen in grey on the left, while the more substantial re-construction of external walls can be seen on the right.

We modelled risk of M. tuberculosis infection during a patient visit of typical duration, the annual risk experienced by a patient undertaking one such visit per month (a ‘chronic patient’) and the annual risk for a nurse working in that space 8 h/day, 200 days/year. The building and proposed retrofits can be seen in Figure 4.

The clinic waiting room had its door and windows fully open during occupied hours, making estimation of the infiltration due to the permeability of the building fabric difficult. The building was therefore modelled as ‘leaky’, with a permeability of 25 m^3^/m^2^/h at 50 Pa applied to external surfaces as cracks at both the top and bottom of the walls.

The results from the CONTAM modelling of the clinic waiting room can be seen in Table 3. They indicate a probability of infection under normal conditions (i.e., existing windows and doors open) of 0.04–0.07% per visit, similar to the 0.05% estimated from the measured CO_2_ data. The extractor fan was ineffective. However, the other retrofits were able to reduce the risk of infection. The most effective low-cost option was the installation of two new windows on opposite sides of the building. This halved the risk of infection. While modelling of the scenario where the windows and doors were closed is highly sensitive to the permeability assumptions, results indicate that, even under the assumption of a ‘leaky’ building, risk of infection per visit increases 50-fold. The replacement of large sections of the walls with latticing and open sections significantly reduced the absolute risk of infection.

**Table 3:**
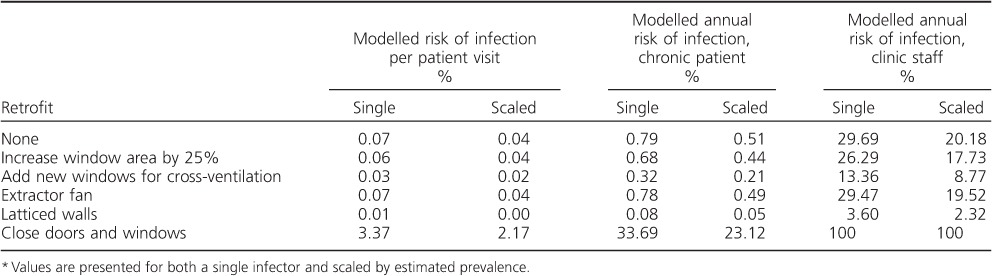
Changes in M. tuberculosis transmission risk following building retrofit, as estimated from CONTAM modelling of building ventilation ^*^

Over a year, building ventilation will vary with weather conditions: for example, wind speed and direction are the primary determinants of cross-ventilation. We estimate this variation in the Appendix. Even during ‘still’ conditions, the new windows and latticing were able to reduce risk by respectively 10% and 70% per hour.

The costs for the retrofits were estimated by a local surveyor to be US$260 to fit a ceiling exhaust, US$330 to fit the two additional windows, US$370 to enlarge the existing windows and US$1200 to replace the bricks with latticework. These costs include labour and materials, but not contracting costs or contractor profit.

## DISCUSSION

Our ventilation measurements suggest that visits to the church and clinic waiting room would carry the highest risk of M. tuberculosis transmission as a result of overcrowding and poor ventilation. However, after accounting for the amount of time spent inside each building over the course of the year, our results indicate that the classroom and the church are potentially the most important locations for M. tuberculosis transmission. We also predict high risks of transmission to patients regularly visiting the clinic and to clinic staff.

The classroom was well designed, with operational windows down either side of the room allowing for high levels of natural ventilation. The predicted M. tuberculosis transmission risk in the classroom was driven by the long periods of time students spend in the space. Conversely, the church and clinic waiting room were poorly designed, leading to a high risk of infection over a much shorter periods of time. To reduce transmission in indoor congregate environments, we should therefore consider intervening in both buildings where occupants spend large amounts of time together as well as in poorly ventilated buildings.

Our empirical estimates of transmission risk assumed a well-mixed indoor space, which may not be reasonable. We assumed homogeneity in infectiousness and susceptibility, a simplifying assumption that might result in transmission in spaces with more occupants being underestimated.^2^

Sensitivity analysis indicated that our estimates of transmission risk were very sensitive to assumptions about infectiousness. Infectious ‘quanta’ were defined, per Riley et al., as ‘the number of infectious airborne particles required to infect [which] may be one or more airborne particles’.^26^ This parameter is best understood at a population rather than at the individual level. As there is no test for reinfection with M. tuberculosis, the impact of previous exposure on M. tuberculosis infection risk is poorly understood. Previous infection provides, at best, incomplete immunity to subsequent infection. Repeated M. tuberculosis exposure might increase the probability that infection is established. Our model assumed that previous M. tuberculosis exposure did not alter subsequent risk of infection.

We also assumed uniform prevalence of TB across non-clinical spaces, which may not be reasonable if illness alters behaviour. Our estimate of prevalence in the clinic was based on a conservative reading of a study from a different part of South Africa.^9^ Despite these limitations, our predicted transmission risks are similar to those observed elsewhere. We predicted annual risks of transmission to health care workers and high school students of respectively 16.9% and 10.6%. As these risks were attributable only to time spent in the clinic waiting room and in the classroom, we may have underestimated the overall risk of infection among health care workers and students. An annual risk of M. tuberculosis infection of 26–27% was described in a cohort of health care workers in Johannesburg.^28^ Annual risks of M. tuberculosis infection comparable to those we predict have been described in adolescents in the Western Cape of South Africa.^29^,^30^

Assuming homogeneity in infectiousness and susceptibility, modelling predicted that retrofits would result in considerable reductions in risk of transmission. However, clinic staff and patients attending regularly would remain at high risk of M. tuberculosis infection. The presumed risk of transmission to health care workers of 100% with the windows closed should be treated with caution, as some individuals are known to be intrinsically less susceptible to M. tuberculosis infection.

Any retrofits should be augmented by administrative controls and the proper use of personal protective equipment. Upper room ultraviolet germicidal irradiation (UVGI) could result in substantial additional reductions in transmission risk and, with ceiling paddle fans, would be effective on still days.^31^

Increasing the rate of natural ventilation may lead to discomfort due to excessively high or low indoor temperatures, energy loss due to conditioned air leaving the building, an increased risk of exposure to vector-borne illnesses, greater risk of forced entry and concerns about privacy. Design considerations should therefore consider alterations to the building that can optimise ventilation without causing reductions in function or comfort.

In conclusion, these results indicate a wide range of transmission risks inside different public buildings in rural South Africa. Churches and clinic waiting rooms were both found to contain high levels of rebreathed air, predicting elevated transmission risk. Predicted risks were also high for students in the well-designed classroom due to the protracted length of time spent indoors. Modelling indicates that low-cost adaptations may dramatically reduce the risk of transmission within buildings, particularly for staff. Our results provide a strong case for intervention studies quantifying the impact on M. tuberculosis transmission of retrofits in congregate settings.^2^

## Figures and Tables

**Figure A.1:**
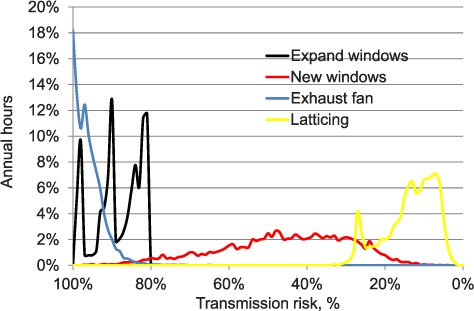
M. tuberculosis transmission risk per hour as a percentage of risk under the default scenario (door and existing windows open). For each adaptation type, this is plotted against the percentage of hours during the year in which that reduction in risk is achieved. The area under the curve for each retrofit sums to 100%.

**Figure A.2:**
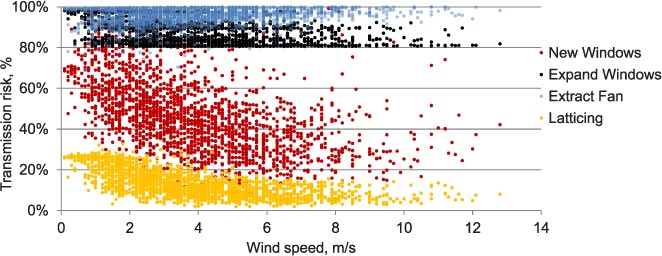
Variation in hourly M. tuberculosis transmission risk with outdoor wind speed, as a percentage of hourly risk under the default scenario (door and existing windows open); each point represents an hour in the year.

**Table A.1:**
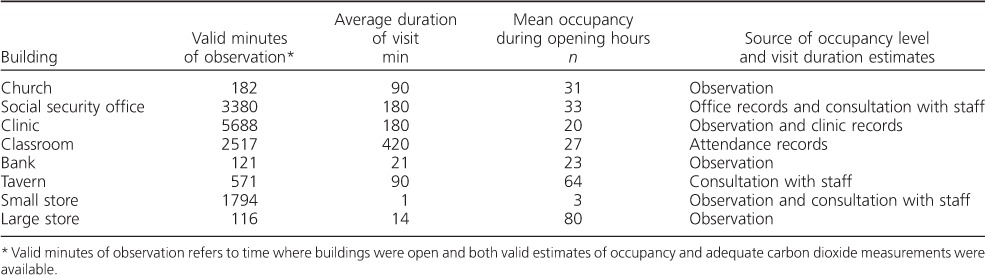
Building observation data: building occupancy and how those data were obtained

**Table A.2:**
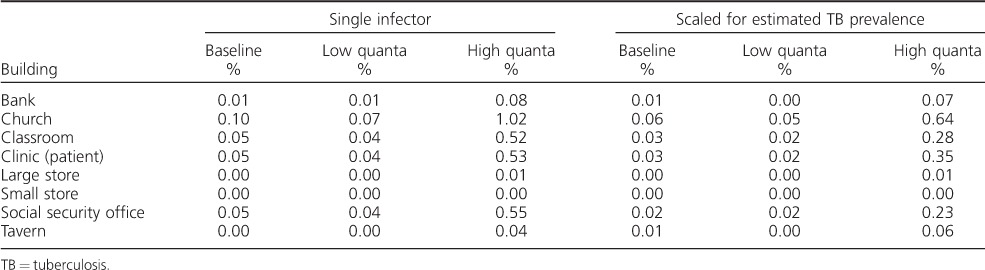
Model sensitivity to assumptions regarding quanta production rates: probability of M. tuberculosis infection per visit

**Table A.3:**
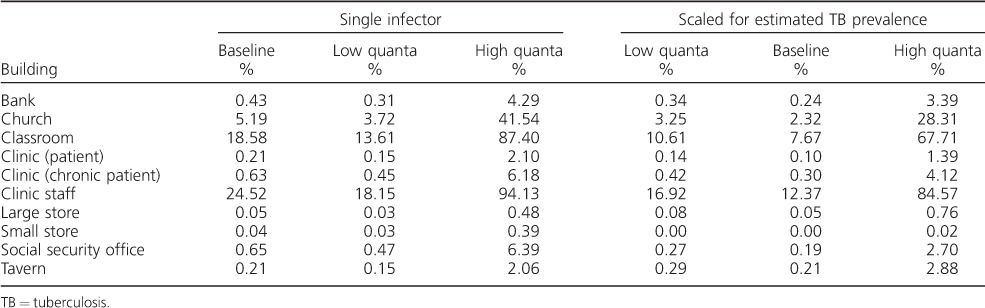
Model sensitivity to assumptions regarding quanta production rates: probability of M. tuberculosis infection per year

**Table A.4:**
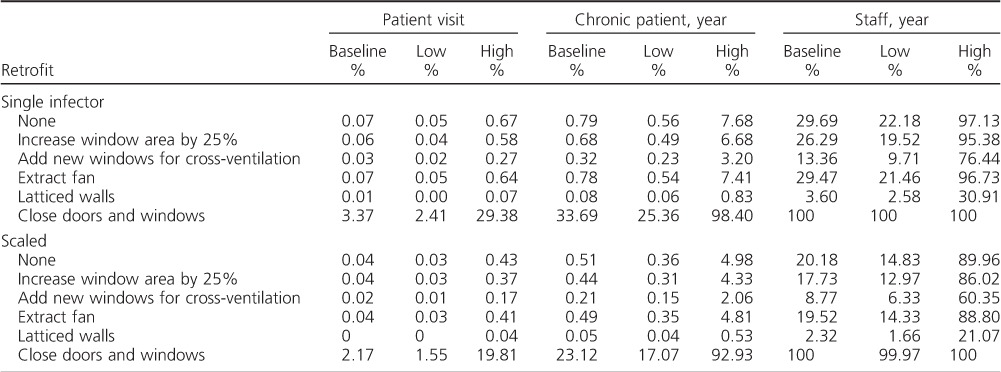
Changes in M. tuberculosis transmission risk following CONTAM modelled retrofits under different assumptions about quanta production rates, for both single infectors and scaled for estimated tuberculosis prevalence

## Footnotes

^*^ The appendix is available in the online version of this article, at http://www.ingentaconnect.com/content/iuatld/ijtld/2016/00000020/00000009/art00008

## APPENDIX

### Estimating risk of infection using the Rudnick and Milton method

The fraction of indoor air comprising exhaled breath was calculated using Equation 1:

where *C* is the measured volume fraction of carbon dioxide (CO_2_) in the indoor air, *C**_O_* is the measured volume fraction of CO_2_ in the outdoor air, and *C**_a_* is the is the volume fraction of CO_2_ added to exhaled breath during breathing, assumed to be 0.038.^1^ The quantum concentration (*N*) (q/m^3^) inside the buildings at each minute was then estimated using Equation 2:

where *I* is the number of infectors, *q* is a quantum generation rate (q/min), *n* is the number of people in the space (obtained from occupancy observations and estimates) and *p* is the respiratory rate. We assumed a baseline *q* of 0.021 q/min^2^ and a respiratory rate of 0.008 m^3^/min.^1^ The mean quanta concentration inside the buildings across the monitoring period was then used to estimate the probability of infection for each occupant using Equation 3:

where *t* is the average exposure time of the occupants, based on observations, interview data and registers. These equations were used to estimate the exposure risks attributable to differences in building performance (e.g., risk per hour of occupation assuming a single infectious individual inside). We further estimate building-specific risks that account for observed average visit durations, and the number of infectious individuals likely to be present:

where *A* is the number of people in the building and *M* is the estimated disease prevalence. We calculate the annual risk as being:

where *x* is the number of visits to the space over the course of a year. The model sensitivity to the rate at which quanta are generated was investigated by recalculating transmission risks using additional ‘low^3^’ and ‘high^4^’ values for quanta generation (respectively 0.89 q/h and 12.6 q/h). It should be noted that these are high and low *average* values from the literature and that much higher rates of quanta production by particularly infectious individuals have been observed.^5^ Model sensitivity to breathing rates was not examined, as this parameter cancels out in the calculation.

### Modelled transmission and external weather conditions

The variation in indoor transmission risk due to weather conditions is shown in Figures A.1 and A.2. The exhaust fan provided a small reduction in risk at low wind speeds, but generally did not offer significant benefit above that provided by the open door and windows. The performance of the other retrofits depended on outdoor wind conditions. However, even during ‘still’ conditions, the new windows and latticing were able to reduce risk by respectively 10% and 70% per hour.

## References

[1] Wood R, Lawn S D, Johnstone-Robertson S, Bekker L. (2011). Tuberculosis control has failed in South Africa – time to reappraise strategy. S Afr Med J.

[2] Yates T A, Tanser F, Abubakar I. (2016). Plan beta for tuberculosis: it's time to think seriously about poorly ventilated congregate settings. Int J Tuberc Lung Dis.

[3] Buu T N, van Soolingen D, Huyen M N T (2010). Tuberculosis acquired outside of households, rural Viet Nam. Emerg Infect Dis.

[4] Glynn J R, Guerra-Assunção J A, Houben R M G J (2015). Whole genome sequencing shows a low proportion of tuberculosis disease is attributable to known close contacts in rural Malawi. PLOS ONE.

[5] Verver S, Warren R M, Munch Z (2004). Proportion of tuberculosis transmission that takes place in households in a high-incidence area. Lancet.

[6] Middelkoop K, Mathema B, Myer L (2015). Transmission of tuberculosis in a South African community with a high prevalence of HIV infection. J Infect Dis.

[7] Andrews J R, Morrow C, Walensky R P, Wood R. (2014). Integrating social contact and environmental data in evaluating tuberculosis transmission in a South African township. J Infect Dis.

[8] Bantubani N, Kabera G, Connolly C (2014). High rates of potentially infectious tuberculosis and multidrug-resistant tuberculosis (MDR-TB) among hospital inpatients in KwaZulu Natal, South Africa indicate risk of nosocomial transmission. PLOS ONE.

[9] Claassens M M, Jacobs E, Cyster E (2013). Tuberculosis cases missed in primary health care facilities: should we redefine case finding?. Int J Tuberc Lung Dis.

[10] Hanifa Y, Fielding K L, Charalambous S (2012). Tuberculosis among adults starting antiretroviral therapy in South Africa: the need for routine case finding. Int J Tuberc Lung Dis.

[11] Gandhi N R, Weissman D, Moodley P (2013). Nosocomial transmission of extensively drug-resistant tuberculosis in a rural hospital in South Africa. J Infect Dis.

[12] Murray E J, Marais B J, Mans G (2009). A multidisciplinary method to map potential tuberculosis transmission ‘hot spots’ in high-burden communities. Int J Tuberc Lung Dis.

[13] Li Y, Leung G M, Tang J W (2007). Role of ventilation in airborne transmission of infectious agents in the built environment? A multidisciplinary systematic review. Indoor Air.

[14] Richardson E T, Morrow C D, Kalil D B, Bekker L-G, Wood R. (2014). Shared air: a renewed focus on ventilation for the prevention of tuberculosis transmission. PLOS ONE.

[15] World Health Organization (2009). WHO policy on TB infection control in health-care facilities, congregate settings and households.

[16] Farley J E, Tudor C, Mphahlele M (2012). A national infection control evaluation of drug-resistant tuberculosis hospitals in South Africa. Int J Tuberc Lung Dis.

[17] Lygizos M, Shenoi S V, Brooks R P (2013). Natural ventilation reduces high TB transmission risk in traditional homes in rural KwaZulu-Natal, South Africa. BMC Infect Dis.

[18] Chamie G, Wandera B, Luetkemeyer A (2013). Household ventilation and tuberculosis transmission in Kampala, Uganda. Int J Tuberc Lung Dis.

[19] Rudnick S N, Milton D K. (2003). Risk of indoor airborne infection transmission estimated from carbon dioxide concentration. Indoor Air.

[20] Andrews J R, Morrow C, Wood R. (2013). Modeling the role of public transportation in sustaining tuberculosis transmission in South Africa. Am J Epidemiol.

[21] Walton G N, Dols W S. (2008). CONTAM 2.4c User Guide and Program Documentation.

[22] Massyn N, Day C, Peer N, Padarath A, Barron P, English R. (2014). District health barometer 2013/14.

[23] Zaidi J, Grapsa E, Tanser F, Newell M-L, Bärnighausen T. (2013). Dramatic increase in HIV prevalence after scale-up of antiretroviral treatment. AIDS.

[24] Gibson D, MacGregor C. (2013). A novel solid state non-dispersive infrared CO2 gas sensor compatible with wireless and portable deployment. Sensors (Basel).

[25] Ayles H, Muyoyeta M, Du Toit E (2013). Effect of household and community interventions on the burden of tuberculosis in southern Africa: the ZAMSTAR community-randomised trial. Lancet.

[26] Riley E C, Murphy G, Riley R L. (1978). Airborne spread of measles in a suburban elementary school. Am J Epidemiol.

[27] Escombe A R. (11 November 2014). What guinea pigs taught me. MTB Transmission 2014.

[28] McCarthy K M, Scott L E, Gous N (2015). High incidence of latent tuberculous infection among South African health workers: an urgent call for action. Int J Tuberc Lung Dis.

[29] Middelkoop K, Bekker L-G, Liang H (2011). Force of tuberculosis infection among adolescents in a high HIV and TB prevalence community: a cross-sectional observation study. BMC Infect Dis.

[30] Andrews J R, Hatherill M, Mahomed H (2015). The dynamics of QuantiFERON^®^-TB Gold In-Tube conversion and reversion in a cohort of South African adolescents. Am J Respir Crit Care Med.

[31] Escombe A R, Moore D A J, Gilman R H (2009). Upper-room ultraviolet light and negative air ionization to prevent tuberculosis transmission. PLOS Med.

